# Supplementary material for: BMP8 and activated brown adipose tissue in human newborns
Source: Nat Commun. 2021 Sep 6;12:5274. doi: 10.1038/s41467-021-25456-z (PMC8421355; doi:10.1038/s41467-021-25456-z)
Supplement: Supplementary file 1 — Supplementary Information [file 41467_2021_25456_MOESM1_ESM.pdf]

Supplementary Information

BMP8 and activated brown adipose tissue in human newborns

Adela Urisarri [1,#], Ismael González-García [2,3,#], Ánxela Estévez-Salguero [2,3], María P. Pata [4], Edward Milbank [2,3], Noemi López [1], Natalia Mandiá [1], Carmen Grijota-Martinez [5], Carlos A. Salgado [6], Rubén Nogueiras [2,3], Carlos Diéguez [2,3], Francesc Villarroya [3,7], José-Manuel Fernández-Real [3,8,9], María L. Couce [1,*] & Miguel López [2,3,*]

[1] Neonatology Service, Department of Pediatrics, University Clinical Hospital of Santiago de Compostela, IDIS, CIBERER, Travesia Choupana, Santiago de Compostela 15706, Spain.
[2] Department of Physiology, CIMUS, University of Santiago de Compostela-Instituto de Investigación Sanitaria, Santiago de Compostela, 15782, Spain
[3] CIBER Fisiopatología de la Obesidad y Nutrición (CIBERobn), 15706, Spain
[4] Biostatech Advice, Training and Innovation in Biostatistics, S.L Santiago de Compostela, 15782, Spain
[5] Department of Cell Biology, Faculty of Biology, Complutense University, Madrid, 28040, Spain.
[6] Instituto Galego de Física de Altas Enerxías (IGFAE), University of Santiago de Compostela, 15782, Spain
[7] Departament de Bioquímica i Biomedicina Molecular, Institut de Biomedicina, Universitat de Barcelona (IBUB), and Institut de Recerca Hospital Sant Joan de Déu, Barcelona 08028, Spain
[8] Institut d'Investigació Biomèdica de Girona (IDIBGI) and Department of Medical Sciences, Faculty of Medicine, University of Girona, Girona, Spain
[9] Department of Diabetes, Endocrinology and Nutrition (UDEN), Hospital of Girona Dr Josep Trueta, Girona, Spain.

#: These authors contributed equally: AU and IG-G

*: Corresponding author:
María L. Couce; Email: Maria.Luz.Couce.Pico@sergas.es
Miguel López; Email: m.lopez@usc.es

Short title: BMP8B and thermogenesis in human newborns

Supplementary Table 1: Body weight change (age 1-age 2)

	Body weight change (age 1-age 2; g)	Body weight change (age 1-age 2; %)
Male Control	-164.4 ± 14.8	-5.1 ± 0.4
Male Cold exposed	-162.9 ± 18.8	-4.8 ± 0.5
Male (all)	-163.5 ± 12.6	-4.9 ± 0.4
Female Control	-175.0 ± 21.4	-5.4 ± 0.6
Female Cold exposed	-152.2 ± 25.4	-4.9 ±0.8
Female (all)	-165.2 ± 16.2	-5.2 ± 0.5
Control (all)	-170.5 ± 13.5	-5.3 ± 0.4
Cold exposed (all)	-158.7 ± 15.4	-4.8 ± 0.4

(Data are expressed as MEAN ± SEM)

Supplementary Table 2. ANOVA table of Linear Mixed Models for Δ temperatures on body and BAT after cold exposure

	Chisq stat	Df	P value	Signif.
Δ Temp				
Intercept	1.105	1	0.2932	
Body/BAT	0.541	1	0.4620	
Control/Cold exposed	12.331	1	0.0004	***
Body/BAT:Control/Cold exposed	6.105	1	0.0135	*

Chisq stat: Chi-squared statistic
Df: degrees of freedom
*P<0.05, ***P<0.001

Supplementary Table 3. ANOVA table of Linear Mixed Models for circulating parameters

Circulating parameter	Chisq stat	Df	P value	Signif.
Glucose				
Intercept	3510.549	1	0.0000	***
Cold exposure	3.358	1	0.0669	
Time	8.346	1	0.0039	**
Cold exposure:Time	1.204	1	0.2726	
TG				
Intercept	1086.388	1	0.0000	***
Cold exposure	0.863	1	0.3530	
Time	83.951	1	0.0000	***
Cold exposure:Time	6.686	1	0.0097	**
T4 (Log)				
Intercept	267.522	1	0.0000	***
Cold exposure	0.083	1	0.7736	
Time	1.267	1	0.2604	
Cold exposure:Time	2.180	1	0.1398	
T3 (Log)				
Intercept	209.773	1	0.0000	***
Cold exposure	0.001	1	0.9819	
Time	1.013	1	0.3142	
Cold exposure:Time	0.984	1	0.3213	
FGF21 (Log)				
Intercept	353.720	1	0.0000	***
Cold exposure	10.333	1	0.0013	**
Time	10.004	1	0.0016	**
Cold exposure:Time	9.230	1	0.0024	**
BMP8B				
Intercept	607.490	1	0.0000	***
Cold exposure	4.271	1	0.0388	*
Time	14.968	1	0.0001	***
Cold exposure:Time	1.088	1	0.2970	

Chisq stat: Chi-squared statistic
Df: degrees of freedom
*P<0.05, **P<0.01, ***P<0.001
Log: logarithm

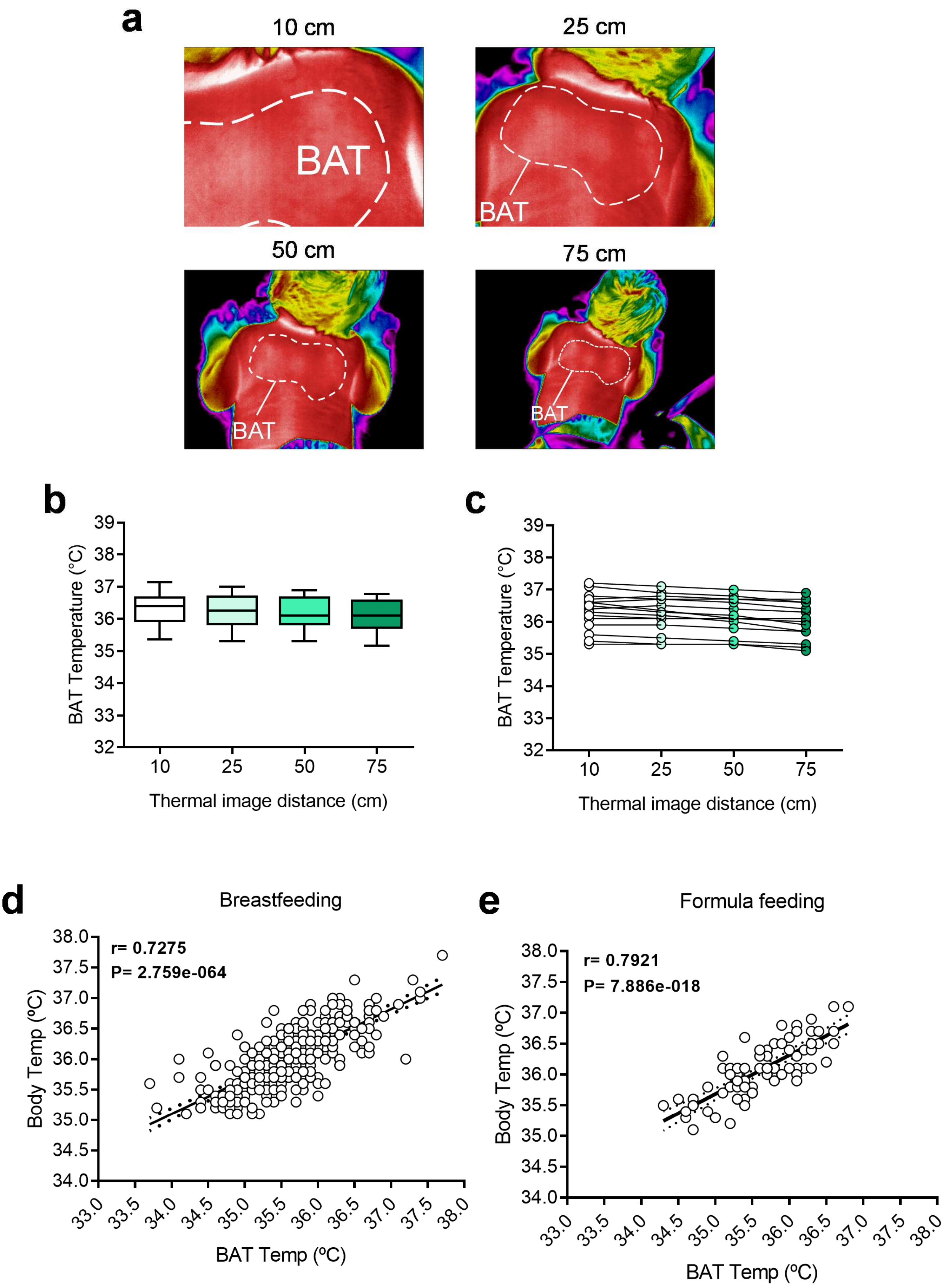

a
10 cm
BAT
25 cm
BAT
50 cm
BAT
75 cm
BAT
b
BAT Temperature (°C)
39
38
37
36
35
34
33
32
10
25
50
75
Thermal image distance (cm)
c
BAT Temperature (°C)
39
38
37
36
35
34
33
32
10
25
50
75
Thermal image distance (cm)
d
Breastfeeding
r= 0.7275
P= 2.759e-064
Body Temp (ºC)
38.0
37.5
37.0
36.5
36.0
35.5
35.0
34.5
34.0
33.0
33.5
34.0
34.5
35.0
35.5
36.0
36.5
37.0
37.5
38.0
BAT Temp (ºC)
e
Formula feeding
r= 0.7921
P= 7.886e-018
Body Temp (ºC)
38.0
37.5
37.0
36.5
36.0
35.5
35.0
34.5
34.0
33.0
33.5
34.0
34.5
35.0
35.5
36.0
36.5
37.0
37.5
38.0
BAT Temp (ºC)

Supplementary Figure 1. Effect of distance of focus on infrared thermography in newborns

- **a.** Representative thermal images of newborns taken at 10, 25, 50 and 75 cm.
- **b.** BAT temperature of human newborns from images taken at 10, 25, 50 and 75 cm. Number of newborns/group (order of groups): 15, 14, 15 and 15. Box plot indicates median (middle line), 25th, 75th percentile (box) and 10th-90th percentiles (whiskers; minima and maxima, respectively).
- **c.** Individual changes in the BAT temperature of newborn from images taken at 10, 25, 50 and 75 cm. Number of newborns/group (order of groups): 15, 14, 15 and 15.
- **d.** Correlation between body temperature and BAT temperature in breastfed newborns. Number of newborns: 381. Association analysis was performed by two-sided Pearson's test; regression line with 95% confidence interval was added when correlation was significant.
- **e.** Correlation between body temperature and BAT temperature in formula-fed newborns. Number of newborns: 76. Association analysis was performed by two-sided Pearson's test; regression line with 95% confidence interval was added when correlation was significant.

Source data are provided as a Source data file.

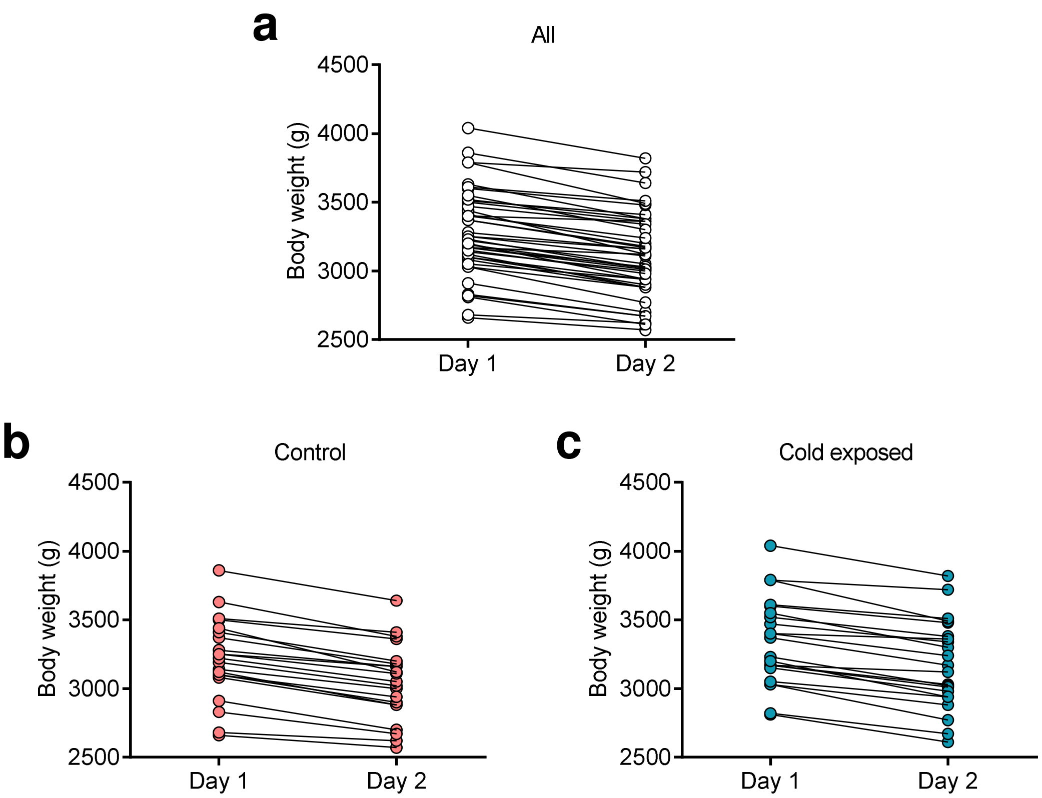

Supplementary Figure 2. Representative individual body weight evolution between day 1 and day 2

- **a.** Control and cold exposed. Number of newborns: 44.
- **b.** Control. Number of newborns: 21.
- **c.** Cold exposed. Number of newborns: 23.

Source data are provided as a Source data file.

a

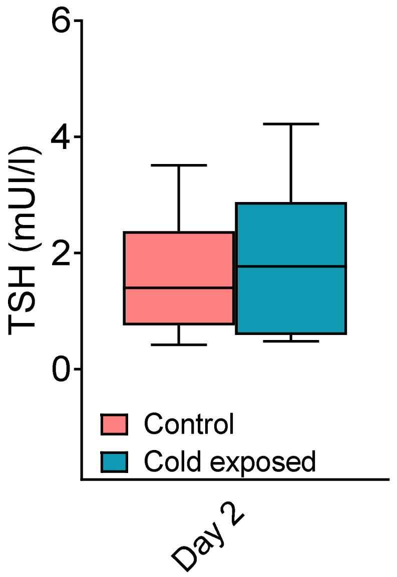

Supplementary Figure 3. Circulating TSH in newborns after cold stimulus at day 2

a. Number of newborns/group (order of groups): 24 and 26. Box plot indicates median (middle line), 25th, 75th percentile (box) and 10th-90th percentiles (whiskers; minima and maxima, respectively). Statistical significance was determined by two-sided Mann-Whitney test. P=0.7932. Source data are provided as a Source data file.

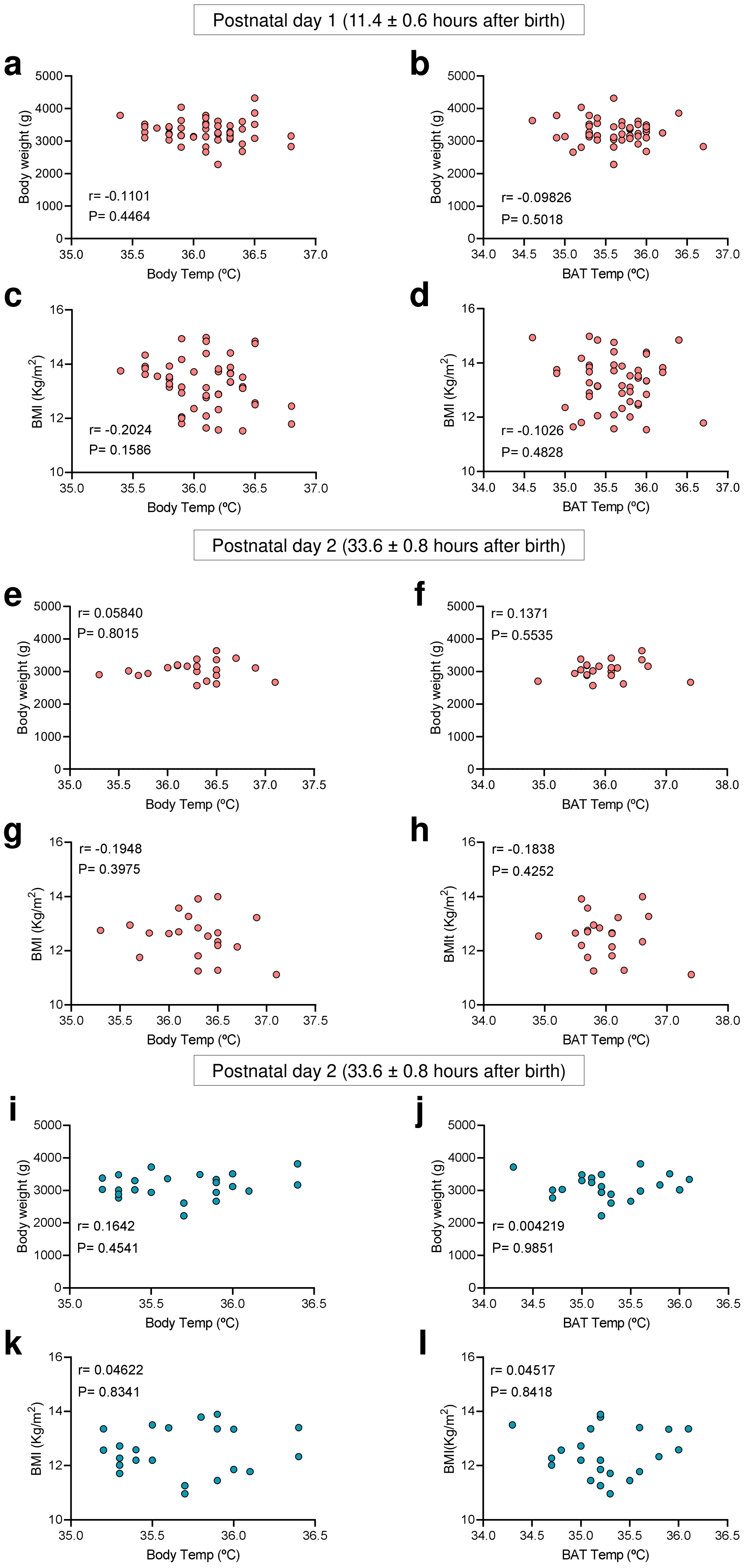

Postnatal day 1 (11.4 ± 0.6 hours after birth)
a
Body weight (g)
Body Temp (ºC)
r= -0.1101
P= 0.4464
b
Body weight (g)
BAT Temp (ºC)
r= -0.09826
P= 0.5018
c
BMI (Kg/m2)
Body Temp (ºC)
r= -0.2024
P= 0.1586
d
BMI (Kg/m2)
BAT Temp (ºC)
r= -0.1026
P= 0.4828
Postnatal day 2 (33.6 ± 0.8 hours after birth)
e
Body weight (g)
Body Temp (ºC)
r= 0.05840
P= 0.8015
f
Body weight (g)
BAT Temp (ºC)
r= 0.1371
P= 0.5535
g
BMI (Kg/m2)
Body Temp (ºC)
r= -0.1948
P= 0.3975
h
BMIt (Kg/m2)
BAT Temp (ºC)
r= -0.1838
P= 0.4252
Postnatal day 2 (33.6 ± 0.8 hours after birth)
i
Body weight (g)
Body Temp (ºC)
r= 0.1642
P= 0.4541
j
Body weight (g)
BAT Temp (ºC)
r= 0.004219
P= 0.9851
k
BMI (Kg/m2)
Body Temp (ºC)
r= 0.04622
P= 0.8341
l
BMI(Kg/m2)
BAT Temp (ºC)
r= 0.04517
P= 0.8418

Supplementary Figure 4. Correlations between body or BAT temperatures and body weight or BMI

Correlation between body temperature **(a, c, e, g, i and k)** and BAT temperature **(b, d, f, h, j and l)** in newborns in control conditions at postnatal day 1 **(a-d)**, postnatal day 2 in control conditions **(e-h)** and postnatal day under cold exposed conditions **(i-l)**. Number of newborns: **a** 50, **b** 49, **c** 50, **d** 49, **e** 21, **f** 21, **g** 21, **h** 21, **i** 23, **j** 22, **k** 23, **l** 22. a. Association analysis was performed by two-sided Pearson's test. Source data are provided as a Source data file.

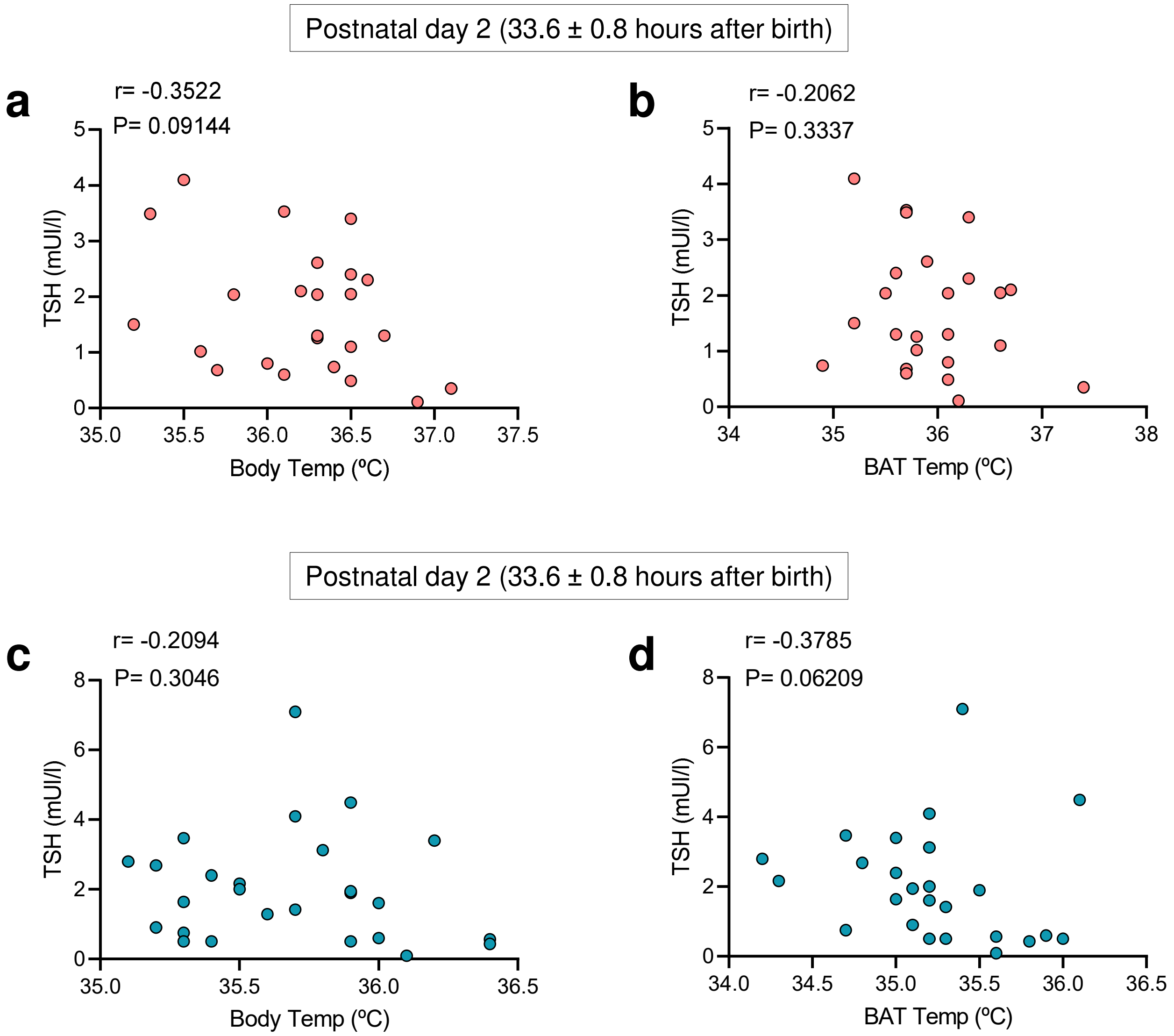

Supplementary Figure 5. Correlations between body and BAT temperatures and circulating TSH in control 2-day old newborns

Correlation between body temperature **(a and c)** and BAT temperature **(b and d)** and circulating TSH in control **(a-b)** and cold exposed **(c-d)** newborns at postnatal day 2. Number of newborns/group: **a** 24, **b** 24, **c** 26, **d** 25. Association analysis was performed by two-sided Pearson's test or Spearman's test. Source data are provided as a Source data file.

a

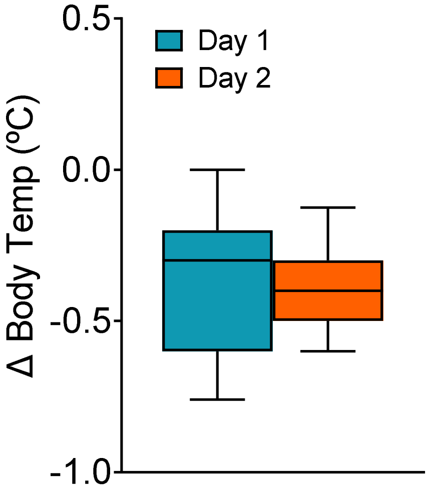

Supplementary Figure 6. Body temperature changes at day 1 and day 2 in cold exposed newborns.

a. Number of newborns/group (order of groups): 23 and 24. Box plot indicates median (middle line), 25th, 75th percentile (box) and 10th-90th percentiles (whiskers; minima and maxima, respectively). Statistical significance was determined by two-sided Mann-Whitney test. P=0.4112. Source data are provided as a Source data file.
