# Supplementary material for: BMP8 and activated brown adipose tissue in human newborns
Source: Nat Commun. 2021 Sep 6;12:5274. doi: 10.1038/s41467-021-25456-z (PMC8421355; doi:10.1038/s41467-021-25456-z)
Supplement: Supplementary file 3 — Reporting Summary [file 41467_2021_25456_MOESM3_ESM.pdf]

nature research

Corresponding author(s): María L. Couce & Miguel López

Last updated by author(s): May 17, 2021

Reporting Summary

Nature Research wishes to improve the reproducibility of the work that we publish. This form provides structure for consistency and transparency in reporting. For further information on Nature Research policies, see our Editorial Policies and the Editorial Policy Checklist.

Statistics

For all statistical analyses, confirm that the following items are present in the figure legend, table legend, main text, or Methods section.

n/a	Confirmed	
☐	☒	The exact sample size (*n*) for each experimental group/condition, given as a discrete number and unit of measurement
☐	☒	A statement on whether measurements were taken from distinct samples or whether the same sample was measured repeatedly
☐	☒	The statistical test(s) used AND whether they are one- or two-sided<br>*Only common tests should be described solely by name; describe more complex techniques in the Methods section.*
☐	☒	A description of all covariates tested
☐	☒	A description of any assumptions or corrections, such as tests of normality and adjustment for multiple comparisons
☐	☒	A full description of the statistical parameters including central tendency (e.g. means) or other basic estimates (e.g. regression coefficient) AND variation (e.g. standard deviation) or associated estimates of uncertainty (e.g. confidence intervals)
☐	☒	For null hypothesis testing, the test statistic (e.g. *F*, *t*, *r*) with confidence intervals, effect sizes, degrees of freedom and *P* value noted<br>*Give P values as exact values whenever suitable.*
☒	☐	For Bayesian analysis, information on the choice of priors and Markov chain Monte Carlo settings
☒	☐	For hierarchical and complex designs, identification of the appropriate level for tests and full reporting of outcomes
☐	☒	Estimates of effect sizes (e.g. Cohen's *d*, Pearson's *r*), indicating how they were calculated

Our web collection on statistics for biologists contains articles on many of the points above.

Software and code

Policy information about availability of computer code

Data collection	Data were collected and analyzed with FLIR Tools Software Package version 1.2.11143.1001.
Data analysis	Data were analyzed with the statistical software GraphPap Prism 8.0.2., R version 4.0.2, emmeans R package version 1.5.1.0005<br>lme4 R package version 1.1-23, WebPower R package version 0.5.2.

For manuscripts utilizing custom algorithms or software that are central to the research but not yet described in published literature, software must be made available to editors and reviewers. We strongly encourage code deposition in a community repository (e.g. GitHub). See the Nature Research guidelines for submitting code & software for further information.

Data

Policy information about availability of data

All manuscripts must include a data availability statement. This statement should provide the following information, where applicable:

- Accession codes, unique identifiers, or web links for publicly available datasets
- A list of figures that have associated raw data
- A description of any restrictions on data availability

The data that support the findings of this study are available from the corresponding author upon reasonable request. The source data underlying Figures and Supplementary Figures are provided as a Source Data file.

Field-specific reporting

Please select the one below that is the best fit for your research. If you are not sure, read the appropriate sections before making your selection.

- [x] Life sciences
- [ ] Behavioural & social sciences
- [ ] Ecological, evolutionary & environmental sciences

For a reference copy of the document with all sections, see nature.com/documents/nr-reporting-summary-flat.pdf

Life sciences study design

All studies must disclose on these points even when the disclosure is negative.

Sample size

A cohort of 50 Caucasian newborns was used for this study. All the subjects were healthy newborns to whom, following the protocol of our center, underwent routine, serial tests at 6-12 hours of life and 24 hours later. Infants with temperature (i.e., fever) or analytical abnormalities were not included in the analyses.

Sample size estimation was based on repeated-measures ANOVA for between-effect test about mean difference among control and intervention groups, considering two time points and assuming that sphericity is met. The significance level alpha and the statistical power were set in 0.05 and 0.80 respectively, and selecting a value for the effect size of 0.40 (large effect size). The total sample size determined was 50 (25 individuals per group). We selected a large effect size due to ethical considerations.

Data exclusions

- Samples were excluded whether their values were outside the 2SD range. This criterion applies to:
One cold exposed individual showed very low BAT temperature values (33.8ºC; < mean-2SD; significant outlier) after stimulation.
One control individual showed a FGF21 value at day 1 of 205.1 pg/ml (> mean + 2SD; significant outlier).
One cold exposed individual showed a FGF21 value at day 2 of 1306.4 pg/ml (> mean + 2SD; significant outlier).

- In the last row of Table 1 and Supplementary Figure 2 the total number of subjects is 44 (rather than 50) because body weight data for day 2 was not available for 6 individuals.

- Criteria were pre-established

Replication

All attempts to replicate the experiments have been successful. Analyses were repeated at twice.

Randomization

Two experimental groups were established (Figure 2A): i) cold exposed newborns to whom a thermogenic stimulus was applied and ii) controls who did not undergo any thermogenic stimulation. Participants were allocated to the control or intervention group by simple random allocation.

Blinding

For clinical reasons, the medical stuff (3 neonatologists) was not blinded during cold exposure protocols since they had to control the newborns' health state during all the procedure. The rest of the clinical staff involved (nurses) was blinded.

Reporting for specific materials, systems and methods

We require information from authors about some types of materials, experimental systems and methods used in many studies. Here, indicate whether each material, system or method listed is relevant to your study. If you are not sure if a list item applies to your research, read the appropriate section before selecting a response.

Materials & experimental systems

n/a	Involved in the study
x	Antibodies
x	Eukaryotic cell lines
x	Palaeontology and archaeology
x	Animals and other organisms
	x Human research participants
x	Clinical data
x	Dual use research of concern

Methods

n/a	Involved in the study
x	ChIP-seq
x	Flow cytometry
x	MRI-based neuroimaging

Human research participants

Policy information about studies involving human research participants

Population characteristics	A cohort of 50 Caucasian newborns was used for this study. Anthropometric parameters [sex, gestational age, body weight, body length, body mass index (BMI), cranial perimeter, age at experimentation and body weight change] from these participants are detailed in Table 1.
Recruitment	All these babies were recruited at the Neonatology Unit of the University of Santiago de Compostela Hospital Complex (CHUS). All of them were healthy newborns to whom, following the protocol of our center, underwent routine, serial tests at 6-12 hours of life and 24 hours later. None of these babies had blood extraction exclusively for this study. Infants with analytical abnormalities were not included in the study. No biases were present.
Ethics oversight	The parents or legal tutors of all newborns gave their written informed consent, which were validated and approved by the Ethical Committee and the Committee for Clinical Investigation of the CHUS (code 2015/079). We certify that all applicable institutional regulations concerning the ethical use of information and samples from human volunteers were followed during this research.

Note that full information on the approval of the study protocol must also be provided in the manuscript.
